# Tiger beetles produce anti-bat ultrasound and are probable Batesian moth mimics

**DOI:** 10.1098/rsbl.2023.0610

**Published:** 2024-05-15

**Authors:** Harlan M. Gough, Juliette J. Rubin, Akito Y. Kawahara, Jesse R. Barber

**Affiliations:** ^1^ McGuire Center for Lepidoptera and Biodiversity, Florida Museum of Natural History, University of Florida, Gainesville, FL 32611, USA; ^2^ Department of Biology, University of Florida, Gainesville, FL 32611, USA; ^3^ Department of Biological Sciences, Boise State University, Boise, ID 83725, USA

**Keywords:** acoustic mimicry, aposematism, Cicindelidae, palatability, tiger moth

## Abstract

Echolocating bats and their eared insect prey are in an acoustic evolutionary war. Moths produce anti-bat sounds that startle bat predators, signal noxiousness, mimic unpalatable models and jam bat sonar. Tiger beetles (Cicindelidae) also purportedly produce ultrasound in response to bat attacks. Here we tested 19 tiger beetle species from seven genera and showed that they produce anti-bat signals to playback of authentic bat echolocation. The dominant frequency of beetle sounds substantially overlaps the sonar calls of sympatric bats. As tiger beetles are known to produce defensive chemicals such as benzaldehyde and hydrogen cyanide, we hypothesized that tiger beetle sounds are acoustically advertising their unpalatability. We presented captive big brown bats (*Eptesicus fuscus*) with seven different tiger beetle species and found that 90 out of 94 beetles were completely consumed, indicating that these tiger beetle species are not aposematically signalling. Instead, we show that the primary temporal and spectral characteristics of beetle warning sounds overlap with sympatric unpalatable tiger moth (Arctinae) sounds and that tiger beetles are probably Batesian mimics of noxious moth models. We predict that many insect taxa produce anti-bat sounds and that the acoustic mimicry rings of the night sky are hyperdiverse.

## 1. Introduction

Insects that fly at night must defend against echolocating bats. This intense selective pressure has helped refine ultrasonically sensitive ears [1], auditory sensors that are capable of providing advance warnings of bat sonar attacks [2]. Bat-detecting ears are found in at least seven orders of insects (Orthoptera, Mantodea, Hemiptera, Coleoptera, Neuroptera, Lepidoptera and Diptera; [3]). In response to hearing the sonar cries of hunting bats, these insects commonly flee or initiate evasive manoeuvers [4,5]. Many moth species take this acoustic battle a step further and produce ultrasonic clicks in response to bat attacks that can startle bat predators [6], signal noxiousness [4], mimic unpalatable models, [7] and jam bat sonar [8]. A recent study revealed that anti-bat ultrasound production is widespread across the lepidopteran phylogeny and around the globe [9]. There are more than 10 independent origins of ultrasound production and Barber *et al*. [9] estimated that perhaps 20% of large-bodied moths (Macroheterocera) produce ultrasound in response to bat sonar attack with the primary function of advertising noxiousness (aposematism) and mimicking unpalatable models.

Several species of tiger beetles (Cicindelidae) in the tribe Cicindelini possess ultrasonically sensitive ears and purportedly produce ultrasound in response to bat attacks [10]. Yager & Spangler [11] showed that some tiger beetles emit ultrasonic clicks as part of a motoric behavioural response when queried with synthetic pulses (40 kHz tones at 100 ms intervals) intended to mimic sonar attack. Upon hearing ultrasound, tiger beetles swing their elytra backwards contacting the leading edge of their beating hindwings producing ultrasonic clicks in time with their wingbeat frequency. These researchers hypothesized that this acoustic response functions as an aposematic warning of chemical protection [11].

Defensive chemicals that tiger beetles produce include benzaldehyde and hydrogen cyanide [12,13]. Benzaldehyde is the most widely studied compound produced by tiger beetles and is known from 13 genera, while hydrogen cyanide has been found in two [14,15]. Using a reduced paradigm, chicks (*Gallus gallus domesticus*) have been shown to reject prey coated with benzaldehyde and hydrogen cyanide [16]. Other work using model tiger beetles has demonstrated that the conspicuous orange abdomens of some beetles, in conjunction with benzaldehyde chemical protection, can reduce the frequency of attacks by robber flies (Asilidae) [17]. We propose that just as bright orange colouring warns visual predators of chemical defence, tiger beetle ultrasonic clicks may be a warning to acoustically specialized bat predators.

Here, we test key hypotheses to understand tiger beetle ultrasound production in the context of bat predation. Authentic echolocation attack sequences have, to our knowledge, never been played back to tiger beetles, yet we hypothesized that beetles would respond to this genuine predatory stimulus with an acoustic response. Furthermore, we predicted that tiger beetles would answer sonar playback with ultrasonic clicks between 30 and 60 kHz, overlapping the most common frequencies used in bat sonar [2]. We hypothesized that tiger beetles are sending an aposematic message to bats and tested the prediction that tiger beetles are unpalatable to these predators. Because we hypothesized tiger beetles are part of a mimicry complex with sound-producing moths, we predicted that the primary temporal and spectral characteristics of tiger beetle warning sounds overlap with sympatric unpalatable tiger moth sounds (Arctinae). Tiger beetles and tiger moths may provide an example of widespread cross-order acoustic mimicry.

## 2. Material and methods

### 2.1. Acoustic playback, recording and analysis

To assay a tiger beetle community for ultrasonic response, we captured individuals of 19 tiger beetle species (seven genera) over two summers in southern Arizona, USA (2017–2018) via hand nets, light traps (a 250 W mercury vapour (MV) bulb and a 15 W florescent blacklight) and pitfall traps. Twelve of these species are diurnal, while seven species are also active nocturnally and can be attracted to artificial light sources [18–21]. We tethered beetles to a thin metal rod affixed to the beetle’s pronotum via dental wax or UV-activated plastic welder (Bondic brand), induced them to fly and then broadcast bat echolocation attack sequences. We placed the speaker behind the flying beetle and the microphone to its side with a distance of 10 cm from the beetle to each, following Barber & Conner [22]. To record beetle sounds, we used Avisoft-RECORDER software on a PC laptop driving an Avisoft D/A (116Hme) sampling at 250 kHz with a CM-16 ultrasonic microphone (approx. ±6 dB from 20 to 110 kHz). We broadcast echolocation attack sequences to flying beetles using Avisoft-RECORDER software on a PC laptop connected to an amplifier with an integrated digital-to-analogue converter (250 kHz sample rate) paired with an ultrasonic speaker (Avisoft UltraSoundGate Player BL Light). Our playback consisted of a series of three echolocation attack sequences. Two of our playback files were recordings of vespertilionid bats with frequency-modulated (FM) sonar: a big brown bat (*Eptesicus fuscus*) and a red bat (*Lasiurus borealis*) attacking moths tethered 10 cm from a microphone in the Sensory Ecology Laboratory flight room (see Barber & Kawahara [23,24] for details). One file was a synthetically generated attack sequence (created in Avisoft-SASLab Pro) based on the short pulse duration, broadband, FM echolocation including certain vespertilionid and most phyllostomid bats [25]. We found nearly identical acoustic responses to playback for all three echolocation attack sequences and hereafter, coded all tiger beetle acoustic responses as responding to bat sonar or not.

We analysed sound files (.wav) using Avisoft-SASLab Pro. Using oscillograms, we manually measured click intervals (from start of a click to start of the next click) and individual click duration. To measure dominant frequency, we quantified (Hann window, 1024 fast Fourier transform) a single click per file with the highest signal-to-noise ratio. To calculate duty cycle, we measured the maximum number of clicks per 100 ms multiplied by the average click duration of three clicks. To compare tiger beetle sounds with sympatric tiger moth sounds recorded from the same geographical location, we used published moth data from Corcoran *et al*. [26]. These data were collected and analysed using the same protocol that we employed to analyse tiger beetle sounds. We assessed the similarity of tiger beetle and sympatric moth anti-bat sounds via a principal component analysis (PCA) run with the base function ‘prcomp’ in R. To visualize the PCA and extract PC1 and PC2 scores, we used the ‘ggbipolt’ and ‘fviz_contrib’ functions in the *factoextra* and *ggplot2* packages, respectively [27,28]. We used ‘adonis2’ from the package *vegan* to compare Procrustes distances between tiger moth and tiger beetle sounds [29].

### 2.2. Palatability to bats

In 2019, we collected eight species of tiger beetles representing five genera in southern Arizona (Pima, Cochise and Santa Cruz counties) using the methods described above. These species were selected because they are common to southern Arizona, all of them are known to produce benzaldehyde, and they all have been assayed for response to bat sonar (table 1). Of these eight species, seven are known to active nocturnally and respond to bat sonar, while only *Cicindelida nigrocoerulea* is strictly diurnal and does not respond to bat sonar. We housed captured tiger beetles, separated by species, in rectangular approximately 2 l plastic containers lined with paper towels at approximately 20°C. To keep the beetles from desiccating, we placed a saturated piece of balled paper towel in each container. We fed the beetles small house crickets (*Acheta domesticus*) and changed the wet paper towel daily.

**Table 1.** Acoustic characteristics of tiger beetles sampled from southern Arizona. (*Cicindelidia punctulata* did not have a high-quality recording to accurately assess dominant frequency. CD, click duration; DC, duty cycle; DF, dominant frequency; ICI, interclick interval.)

species	benzaldehyde production	*n*	ICI (ms)	CD (ms)	DC	DF (kHz)
*Brasiella wickhami*	yes^a^	1	12.48	0.26	2.08	59
*Cicindelidia ocelata*	yes^b^	1	12.57	0.43	3.12	35
*Cicindelidia sedecimpunctata*	yes^b^	2	12.73 ± 1.07	0.24 ± 0.04	1.97 ± 0.2	34.5 ± 0.71
*Jundlandia lemniscata*	yes^a^	4	12.04 ± 0.75	0.18 ± 0.02	1.67 ± 0.19	44 ± 5.57
*Cicindelidia punctulata*	yes^a^	2	13.83 ± 0.2	0.4 ± 0.14	3.16 ± 1.15	—
*Ellipsoptera marutha*	yes^a^	3	15.17 ± 1.07	0.23 ± 0.03	1.37 ± 0.11	57.5
*Eunota fulgoris*	yes^a^	5	15.18 ± 1.09	0.38 ± 0.09	2.71 ± 0.65	42.5 ± 6.14

^a^ Presence of benzaldehyde was sourced from Kelly & Shilling [15].

^b^ Presence of benzaldehyde was sourced from Pearson [17].

We collected scarab beetles (*Phyllophaga* sp.) using a MV light trap in southern Arizona as controls in our palatability experiments. We chose to use adult scarabs in addition to mealworms (*Tenebrio molitor* larvae) as controls because they have been shown to be consumed by the big brown bat (*E. fuscus*) [30] and because they more closely resemble sclerotized tiger beetles. We used mealworms owing to their known palatability from previous studies with bats [24,31]. We housed scarabs as we did tiger beetles.

For palatability trials, we captured adult big brown bats (*E. fuscus*) in Idaho, USA using mist nets in the field and by capturing roosting bats. Big brown bats are distributed across North and South America and overlap with tiger beetles and moths in Arizona [32]. Our collection and housing of bats was in accordance with IACUC protocols (AC19-012) and standard operating procedures in the Sensory Ecology Laboratory at Boise State University, Idaho. We used five bats of both sexes (one male and four females) to test tiger beetle palatability. We maintained bats for two months and fed them a vitamin-fortified mealworm diet and gave them unrestricted access to water.

To test tiger beetles’ palatability to bats*,* we trained big brown bats to eat mealworms from forceps while being held in a gloved hand. Our experimental protocol consisted of offering a control, followed immediately by an experimental tiger beetle, then a second control. We randomized presentation order of control and experimental species and scored palatability using a 0–6 scale [31,33]. For each insect, we recorded the following scores if the body part was consumed: head = 1, thorax = 2 and abdomen = 3. A completely consumed insect was given a score of 6, while one that had its thorax and abdomen consumed but not its head had a score of 5. To compare palatability between each experimental species and the controls, we used a two-sample Kolmogorov–Smirnov test [34], with 0.05α and Bonferroni correction implemented in R [35].

## 3. Results

**Figure 1.**
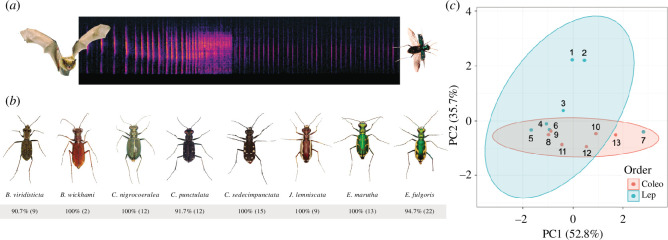
Tiger beetles produce anti-bat ultrasound. (*a*) Spectrogram of a played-back echolocation attack with ultrasonic response produced by a tethered *Ellipsoptera marutha*. (*b*) Percentages indicate palatability to big brown bats (*Eptesicus fuscus*; see §2). Numbers in parenthesis are sample sizes for palatability trials. (*c*) Principal component analysis (PCA) plot of tiger beetle sounds and sympatric chemically defended tiger moth sounds. Tiger beetle sounds are nested within the acoustic space of tiger moth warning signals. The following species are associated with the numbered points on the PCA: 1, *Bertholdia trigona*; 2, *Carales arizonensis*; 3, *Cisthene tenufaschia*; 4, *Cisthene martini*; 5, *Pygarctia rosciapitis*; 6, *Euchates antica*; 7, *Ctenucha venosa*; 8, *Ellipsoptera marutha*; 9, *Brasiella wickhami*; 10, *Eunota fuigoris*; 11, *Jundlandia lemniscata*; 12, *Cicindelidia sedecimpunctata*; 13, *Cicindelidia ocelata*. See §3 for details.

We broadcast bat echolocation attack sequences to 19 tiger beetle species and reported anti-bat sound production (see figure 1*a*) by seven species (table 1) (see the electronic supplementary material, files S1 and sound files in S2). The tiger beetle community’s sounds had an average dominant frequency of 43.1 ± 8.57 kHz (range 59–35 kHz), an average interclick interval of 13.79 ± 1.62 ms, an average click duration of 0.28 ± 0.09 ms and an average duty cycle of 2.16 ± 0.64% (see the electronic supplementary material, files S2 and S3).

To compare anti-bat sounds of sympatric (southern Arizona) tiger beetles and tiger moths, we used moth data from Corcoran *et al*. [26] (see the electronic supplementary material, file S4) and conducted a PCA analysis of three sound parameters collected from both beetles and moths (click duration, duty cycle and dominant frequency). PC1 explained 52.8% of the variation in the data and was predominantly composed of pulse duration and dominant frequency, while PC2 explained 35.7% of the variation and was predominantly defined by pulse duration (figure 1*c*). Visualization of 95% confidence levels for multivariate *t*-distributions (depicted via ‘stat_elipse’ in *ggplot* and coloured ellipses in figure 1*c*) show clear overlap between beetle and moth sounds. Our analysis of variance between tiger beetle and tiger moth sounds indicated that these groups did not statistically differ (*p* >0.05; see R script for test and outputs in the electronic supplementary material, file S6). Thus, their ultrasonic sounds are probably similar to the ears of a bat.

To determine if this purported mimicry between tiger beetles and tiger moths was Müllerian [36] or Batesian [37], we tested the palatability of each tiger beetle species against big brown bats (*E. fuscus*). We conducted 94 palatability trials in which 90 tiger beetles from eight species were completely consumed by big brown bats (95.7%), two tiger beetles were rejected and two were partially consumed (figure 1*b*; table 1; electronic supplementary material, file S5). All tiger beetle species in this study were palatable to *E. fuscus* (*p* < 0.006; table 1). Furthermore, both controls were palatable (scarabs, 82.2%, *n* = 46, *p* < 0.014; mealworms, 100% *n* = 143, *p* < 0.001).

## 4. Discussion

Tiger beetles respond to broadcast of authentic bat echolocation attack sequences with ultrasound production. All seven species that answered bat sonar with ultrasonic clicks are known to be active nocturnal fliers attracted to collecting lights [18–20]. Of the 12 species that did not respond to bat sonar, all are strictly diurnal and not attracted to lights, with the exception of one species (*Cicindelidia hemorrhagica*) that has occasionally been collected at lights [20]. Ultrasound production has been reported to be a plastic behaviour that has originated only in nocturnally active lineages that have experienced the powerful selective pressure of echolocating bats [18–20,38], and we confirm this result. Further evidence indicating that tiger beetles are signalling bats comes from the close match between the peak frequency of the tiger beetle sounds we recorded and the peak sonar frequency of sympatric bats (beetles: 43.1 ± 8.6 kHz; bats: 39.8 ± 14.2 kHz (mean ± s.d.)) [39].

Tiger beetles produce clicks in time with their wingbeat cycle as their hindwings percuss against the elytra and are necessarily low duty cycle (percentage of time occupied by sound; 1–3%). These simple sounds are therefore unlikely to have a sonar jamming function as has been shown for some moth sounds of higher duty cycle (15+%; [8,9,24,40]). A highly plausible alternative is that tiger beetles are advertising noxiousness acoustically. To test this possibility, we captured big brown bats (*E. fuscus*) and offered them eight different tiger beetle species (94 individuals) in handheld feeding trials. Almost all individuals of each tiger beetle species we tested were consumed by bats (approx. 95%), indicating that the advertisement of unpalatability cannot be the function of these echolocation-elicited sounds. Instead, tiger beetles are probably mimicking the sounds of sympatric, noxious tiger moths.

To assess a key prediction of this mimicry hypothesis, we compared sounds of tiger beetles to a sympatric community of primarily unpalatable tiger moths [8,41]. Using PCA, we compared acoustic characteristics of both groups and found that beetle sounds are nested within the acoustic space of moth warning signals (figure 1*c*). Tiger beetles are quite likely Batesian mimics of ultrasound-producing moths. It is interesting that while all tiger beetles tested produce benzaldehydes [14,15], they are not chemically defended against bats. Perhaps, the concentration of benzaldehydes in these species is not sufficient to deter bat predators or benzaldehyde production is targeted at invertebrate predators such as ants and robber flies [17,42]. Other tiger beetle species that we did not test here are capable of producing hydrogen cyanide, which may defend them against vertebrates [13].

Tiger beetles now join ultrasound-producing moths from seven families that respond to bat echolocation attack with ultrasound and are palatable to bats (Geometridae [41], Sphingidae [24], Crambidae, Erebidae, Noctuidae, Notodontidae and Pyralidae [9,24,41]). These animals are probably Batesian mimics of noxious model moths [9]. Barber & Conner [7] demonstrated that bats naïve to ultrasound-producing tiger moths easily learn to avoid noxious acoustic models and generalize this avoidance learning to other tiger moth species that are either chemically protected (Müllerian) or not (Batesian) [7]. Definitive evidence for Batesian mimicry between tiger moths and tiger beetles awaits similar behavioural experiments.

It is evident that tiger beetle ultrasound production is an incredibly labile trait found throughout the tribe Cicindelini [21,38]. Nocturnal flight, and thus exposure to bat predation, seems to be a powerful predictor of acoustic response. While we do not have the taxon sampling for inference concerning the number of origins of this trait, all available data show that anti-bat sound production in tiger beetles is limited to Cicindelini, a group with a crown age of (approx. 48 ± 10 Ma [21,43]) around the same time that tiger moths, the purported acoustic model, arose (approx. 50 ± 10 Ma [1,9]). These dates closely align with the origin of laryngeal echolocation in bats (approx. 50 ± 10 Ma [44,45]) and suggest that these predators may have been driving cross-order acoustic mimicry for millions of years. Adding tiger beetles to the now substantial list of moths that acoustically respond to bat sonar [9] means that acoustic mimicry is even more phylogenetically broad than previously thought. We predict that many additional insect taxa will be shown to produce similar anti-bat sounds, and that acoustic mimicry rings of the night sky occur worldwide and are phylogenetically diverse.

## Data Availability

Electronic supplemental materials for appendices S1 and S3–S6 can be found online [46]. Appendix S2 can be found on Dryad [47]. Appendix S1. Arizona tiger beetle response to simulated bat attack and nocturnal activity. Appendix S2. Acoustic recording (.wav) of tiger beetle response to simulated bat attack. Appendix S3. Summary of acoustic parameters of tiger beetle response to simulated bat attack (See methods for description). Appendix S4. Summary of acoustic parameters of tiger moths response to simulated bat attack from published moth data in Corcoran *et al*. [26]. Appendix S5. Tiger beetle palatability to big brown bats (see methods for description). Appendix S6. R script for test and outputs of PCA analysis.
